# Prepectoral Breast Reconstruction: Early and Long-Term Complications and Outcomes of Total Coverage Acellular Dermal Matrix and Implants Vs Polyurethane-Coated Implants Without Use of Acellular Dermal Matrix

**DOI:** 10.1093/asj/sjaf158

**Published:** 2025-08-08

**Authors:** Marzia Salgarello, Mauro Barbera, Giuseppe Visconti, Lorenzo Scardina, Gianluca Franceschini, Alba Di Leone, Liliana Barone Adesi, Nicolò Lentini, Roberta Pastorino

## Abstract

**Background:**

Immediate prepectoral breast reconstruction has emerged as a prominent alternative to subpectoral techniques, offering favorable outcomes in selected patients. Among available options, implant coverage with acellular dermal matrix (ADM) and the use of polyurethane (PU)-coated implants without ADM represent 2 widely adopted strategies.

**Objectives:**

The aim of the authors this study is to examine the comparative efficacy and complication profiles of implant coverage with ADM and the use of PU-coated implants without ADM.

**Methods:**

This retrospective cohort study included 97 patients (135 breasts) undergoing immediate prepectoral breast reconstruction following nipple-sparing, skin-sparing, or skin-reducing mastectomy between April 2015 and October 2019. Patients were stratified into 2 groups: those receiving ADM-covered textured implants and those receiving PU-coated implants. Outcomes assessed included early (<4 weeks), mid-term (>4 weeks), and long-term (≥1 year) complications, as well as aesthetic results evaluated through blinded assessment using a standardized Likert scale.

**Results:**

PU-coated implants were associated with significantly lower rates of early postoperative seroma (2.9% vs 33.8%, *P* < .001) and infection (1.4% vs 6.2%). At 5 years, the incidence of severe capsular contracture (Baker Grade 3-4) was markedly higher in the ADM group (47.7% vs 24.3%, *P* < .001), particularly in patients who had not received postmastectomy radiotherapy. No significant differences were observed in the incidence of rippling or step-off deformities. Aesthetic outcomes were superior in the PU group, with significantly better breast symmetry and global aesthetic evaluation (*P* = .021).

**Conclusions:**

PU-coated implants offer a safer and more effective approach in immediate prepectoral breast reconstruction, with reduced complication rates and improved aesthetic outcomes compared with ADM-covered implants. Patient-specific anatomical and oncologic factors should guide implant selection to optimize surgical outcomes.

**Level of Evidence: 4 (Therapeutic):**

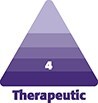

Breast reconstruction following mastectomy has been a significant focal point of reconstructive plastic surgery for over 50 years. In Italy, more than 55,000 new cases of breast cancer were diagnosed in 2022.^1^ Among reconstructive techniques, implant-based reconstruction is widely utilized. It is estimated that over 15,000 implants are placed annually for reconstructive purposes in Italy.^2^

In recent years, there has been a growing trend to consider immediate prosthetic breast reconstruction (IPBR) as an integral part of the surgical treatment following conservative mastectomies, including nipple-sparing mastectomy (NSM), skin-sparing mastectomy (SSM), and skin-reducing mastectomy (SRM). To minimize complications associated with traditional subpectoral implant-based reconstruction—such as animation deformity, postoperative pain, and shoulder dysfunction—many breast surgery units have shifted toward prepectoral reconstruction. This approach enables direct-to-implant reconstruction even in large and ptotic breasts, with low complication rates and excellent aesthetic outcomes.^3-5^

Since the adoption of this approach, 2 main techniques for implant placement have been described: the use of acellular dermal matrices (ADMs) to cover the implant and the use of polyurethane (PU)-coated implants without ADM. The literature reports that both approaches achieve excellent aesthetic results. The use of interfaces in prepectoral techniques builds on experience gained from dual-plane approaches, where such materials proved essential in ensuring proper positioning, providing structural support, and concealing the implant beneath the skin. Many authors continue to advocate for the use of ADM in various configurations, emphasizing its benefits in improving pocket definition and adding a protective layer to the soft tissues.^6,7^ However, some studies suggest that reconstruction with ADM-covered implants increases the risk of seroma formation and incurs higher costs, whereas reconstruction with PU-coated implants is associated with a higher incidence of rippling.

Despite the numerous aesthetic advantages attributed to the use of interfaces in prepectoral reconstruction, one of their primary goals remains the prevention of capsular contracture. Biological ADM may achieve this by isolating the implant and reducing the body's inflammatory response. Conversely, synthetic PU stimulates a controlled inflammatory response, which inhibits effective myofibroblast contraction and promotes less organized collagen deposition.^8,9^ Moreover, PU-coated implants have shown a reduction in contracture rates, even in postradiotherapy patients undergoing 2-stage breast reconstruction.^10^ Although substantial evidence supports the efficacy of PU-coated implants, comparative analyses between the 2 techniques remain limited. Additionally, the use of PU-coated implants has demonstrated reduced contracture rates in postradiotherapy breast reconstruction in 2-stage procedures.^11-21^

The aim of the authors of this study is to compare the short-, medium-, and long-term outcomes of direct-to-implant prepectoral reconstruction using textured implants with ADM for total implant coverage vs PU-coated implants without ADM. The authors will evaluate the maintenance of aesthetic outcomes over time and compare capsular contracture rates, even after postmastectomy radiotherapy (PMRT).

## METHODS

This retrospective study involved patients who underwent immediate breast reconstruction with prepectoral implants after NSM, SSM, and SRM between April 2015 and October 2019. Patients were divided into 2 main groups.

The PU group received PU-coated implants (Replicon/Opticon Microthane implants, POLYTECH Health & Aesthetics GmbH, Dieburg, Germany), and the ADM group received textured implants (Polytech Replicon/Opticon POLYtxt) with total coverage ADM (Braxon; DECOmed, Venice, Italy).

The 2 reconstructive approaches were performed sequentially: initially, reconstructions were carried out using ADM and textured implants; subsequently, this approach was discontinued in favor of the exclusive use of PU-coated implants. As a result, patients in the ADM group had a longer follow-up period compared with those in the PU group. Nevertheless, for the purposes of this study, all patients were evaluated at comparable postoperative time points, as detailed in the following section.

All breast reconstructions were performed following mastectomy, which was carried out by breast surgeons as the first surgical step. The indication for the type of mastectomy—SSM or NSM—was determined by the breast surgeons based on oncological criteria and individual patient characteristics. The reconstructive procedures were then conducted by 3 experienced plastic surgeons, all of whom adhered to standardized surgical protocols.

Inclusion criteria encompassed patients undergoing therapeutic or prophylactic mastectomy with immediate reconstruction. Known risk factors for complications, such as obesity, active smoking, and diabetes mellitus (DM), were not considered exclusion criteria. Patients with a history of previous breast-conserving surgery and radiotherapy were excluded. Complication data were collected per operated breast, whereas aesthetic outcome data were collected per patient.

Intraoperatively, mastectomy flap thickness was assessed at the surgical incision margins at 4 distinct points—2 along the superior margin and 2 along the inferior margin. Flaps were deemed suitable for prepectoral reconstruction if the measured thickness was ≥1 cm and the tissue demonstrated uniform consistency on palpation, with no visual evidence of cutaneous compromise. In all patients, flap perfusion was evaluated using indocyanine green (ICG) angiography (Fluobeam, Fluoptics, Grenoble, France).^22^ Following intravenous bolus administration of ICG, perfusion was assessed using the Fluobeam system in real time. The evaluation included all phases of perfusion: the early arterial phase (initial appearance of fluorescence), the venous phase (distribution of the dye), and the washout phase (clearance of the signal). Quantitative analysis of fluorescence intensity was performed by comparing different areas of the flap to ensure adequate and uniform perfusion, and all intraoperative decisions regarding tissue viability were based on both quantitative imaging and clinical assessment.

Following this evaluation, meticulous hemostasis of the prepectoral pocket was performed. Sizers were used to verify shape and volume before placing the definitive implant. In all patients, bruised wound edges were removed before closure to optimize the healing process.

In all cases, before implant placement, sterile gloves were changed, implants were rinsed in antibiotic solution (sodium rifamycin), and the breast skin was disinfected with povidone-iodine. For PU-coated implants, particular attention was paid to the correct implant positioning, making sure that the edges of the implant adhered well to the underlying muscle and did not fold over into the surrounding tissues.

Reconstructions using total implant coverage with ADM and textured implants were performed following the manufacturer's recommendations.

This study did not require approval by an IRB. Nevertheless, written informed consent was obtained from all included patients, and the study was conducted in accordance with the ethical principles outlined in the Declaration of Helsinki.

### Surgical Technique

The mastectomy incision was preferably made along the lateral radial area, especially if large and/or ptotic breasts. As a second option, the incision was made in the inframammary fold (IMF; small/medium size and nonptotic breast). In other cases, that is, scars after previous surgeries, the approach was evaluated based on the patient's specific needs.

Preoperative antibiotic prophylaxis with cefazolin was administered and continued until the drains were removed.

Two Jackson–Pratt drains were placed laterally in the IMF for each breast. Internally, 1 was placed above the implant and the other below the implant. Drains were removed when the daily output was <25 mL for 2 consecutive days. After drain removal, all patients underwent an ultrasound at 1 week and weekly for 1 month to assess any fluid collections.

On the first postoperative day, patients were provided with a soft bra, which they were instructed to wear continuously for at least 1 month. Patients were advised to perform daily dressing changes.

Postoperative complications were classified as “short-term” if they occurred within 4 weeks of surgery and “mid-term” if they occurred or persisted after the 4-week postoperative period. This distinction was made to assess the behavior and integration of the implants beyond the immediate postoperative period, including complications that arose within the first month but persisted. Seromas were defined as fluid collections requiring aspiration, whereas a thin film of periprosthetic fluid was not classified as seromas. Seromas or fluid collections were assessed by means of ultrasonography. Infections were defined as cases presenting with local redness, fever, and/or an increase in C-reactive protein levels not attributable to causes other than breast reconstruction.

Long-term complications (occurring after 1 year) were analyzed: capsular contracture, rippling, step-off deformity, thinning/dyschromia, minus areas/adhesions, and breast implant-associated anaplastic large cell lymphoma (BIA-ALCL).

The degree of capsular contracture was assessed using the 5-point Baker–Spear classification (1a-4) by a single blinded plastic surgeon at 3 and 5 years post surgery. Similarly, the degree of “rippling” was assessed using the 4-point Vidya classification (1-4) to measure the severity of visible or palpable wrinkling during the 1-year follow-up, along with the appearance of step-off deformity. Grades 3 and 4 were considered “clinically visible rippling.”^23,24^

The postoperative aesthetic evaluation of the reconstructed breast was performed by a group of 2 independent plastic surgeons, who assessed the outcomes blindly using a 3-point Likert scale (0, 1, and 2). Evaluation parameters included volume, shape, position of the nipple–areola complex (NAC), visibility of implant edges, and symmetry with the contralateral breast. The choice of a 3-point Likert scale (0 = poor, 1 = acceptable, and 2 = good) was made to simplify the evaluation process in a retrospective context and to facilitate consistent scoring based on standardized photographic documentation. This scale has proven to be practical and reproducible in assessing key outcomes such as volume, contour, NAC position, implant edge visibility, and symmetry.

Aesthetic assessments were conducted independently by 2 experienced plastic surgeons, both blinded to the type of implant and treatment group. In cases where discrepancies arose between the 2 raters, a consensus was reached through a joint re-evaluation and discussion of the photographic material.

### Statistical Methods

Continuous variables were summarized as mean and standard deviation or median and interquartile range (IQR), depending on their distribution. Categorical variables were described using absolute frequencies and percentages. Comparisons between implant groups (ADM vs PU) were performed using Student's *t*-test or the Mann–Whitney *U* test, as appropriate. For categorical variables, Pearson's χ^2^ test or Fisher's exact test was applied.

The association between capsular contracture (Grade 1-2 vs Grade 3-4) and implant type, as well as PMRT, was assessed using logistic regression models adjusted for age, BMI, mastectomy volume, implant volume, and mastectomy and flap thickness.

Additionally, the relationship between rippling grade (Grade 1-2 vs Grade 3-4) and implant type, thickness, age, BMI, mastectomy volume, and implant volume was analyzed using logistic regression models.

For the ordinal aesthetic outcomes, ordinal logistic regression models were employed to assess their association with implant type, adjusting for age, BMI, and bilateral interventions. The parallel line (proportional odds) assumption was verified using the Brant test, which confirmed its validity (*P* ≥ .05).

Effect sizes were reported as odds ratios (ORs) with corresponding 95% CIs. Statistical significance was set at a 2-sided *P*-value <.05.

All analyses were conducted using R software (version 4.4.0, released on April 24, 2024) for Windows.

## RESULTS

### Patient Characteristics

A total of 97 patients (135 breasts) underwent immediate breast reconstruction using the prepectoral technique between April 2015 and October 2019. The average patient follow-up time was 40 months, ranging from 12 to 60 months. Fifty-four patients (70 breasts) received PU-coated implants (Group A), whereas 43 patients (65 breasts) received textured implants and total cover ADM (Group B).

The median age of the patients was 47.8 years (IQR, 43.4-54.7), with a median BMI of 23.3 (IQR, 21.1-25.7). No statistically significant differences were observed in the demographic characteristics between the 2 groups (Table 1).

**Table 1.** Baseline Patient and Breast Characteristics in the 2 Groups

Median (q1-q3)/*n* (%)	Overall	PU	ADM	*P*-value
Patients	97	54	43	
Breast	135	70	65	
Bilateral	38 (39.2)	16 (29.6)	22 (51.2)	.031
Age (years)	47.8 (43.4-54.7)	47.1 (42.5-51.5)	49.1 (44.8-58.2)	.109
BMI	23.3 (21.1-25.7)	22.7 (20.8-25.9)	23.9 (22.6-25.5)	.218
Smoking status				.709
Never	77 (79.4)	43 (79.6)	34 (79.1)	
Former	11 (11.3)	7 (13.0)	4 (9.3)	
Current	9 (9.3)	4 (7.4)	5 (11.6)	
Tumor type				.506
DCIS	19 (19.6)	11 (20.4)	8 (18.6)	
IDLC	1 (1.0)	1 (1.9)	0 (0.0)	
IDC	68 (70.1)	39 (72.2)	29 (67.4)	
ILC	4 (4.1)	2 (3.7)	2 (4.7)	
LCIS	5 (5.2)	1 (1.9)	4 (9.3)	
DM 1	1 (1.0)	0 (0.0)	1 (2.3)	.443
DM 2	3 (3.1)	2 (3.7)	1 (2.3)	1.000
Chemotherapy				<.001
No	56 (57.7)	20 (37.0)	36 (83.7)	
Preop	33 (34.0)	30 (55.6)	3 (7.0)	
Postop	8 (8.2)	4 (7.4)	4 (9.3)	
Mastectomy type				.263
NSM	82 (84.6)	45 (83.3)	37 (86.0)	
SRM	4 (4.1)	1 (1.9)	3 (7.0)	
SSM	11 (11.3)	8 (14.8)	3 (7.0)	
Contralateral surgery^a^	31 (52.5)	19 (50.0)	12 (57.1)	.560
PMRT	25 (18.5)	22 (31.4)	3 (4.6)	<.001
Implant volume	360 (265-420)	295 (235-400)	375 (325-450)	<.001
Mastectomy volume	285 (186-420)	248 (183-364)	320 (214 -502)	.006
Mastectomy flap thickness (continuous)	1.0 (0.9-1.2)	1.10 (0.9-1.2)	1.0 (0.8-1.0)	<.001
Mastectomy flap thickness (categorical)				.091
<1	41 (30.4)	18 (25.7)	23 (35.4)	
1-1.49	83 (61.5)	43 (61.4)	40 (61.5)	
≥1.5	11 (8.1)	9 (12.9)	2 (3.1)	

ADM, acellular dermal matrix; DCIS, ductal carcinoma in situ; DM, dermal metastases; IDC, invasive ductal carcinoma; IDLC, invasive ductolobular carcinoma; ILC, invasive lobular carcinoma; NSM, nipple-sparing mastectomy; PMRT, postmastectomy radiotherapy; PU, polyurethane; SRM, skin-reducing mastectomy; SSM, skin-sparing mastectomy. ^a^In unilateral patients.

### Short- and Medium-Term Complications

#### Seroma Formation

The incidence of postoperative seroma in the PU group was 2 cases (2.9%), and in the ADM group, it was 22 cases (33.8%). At mid-term follow-up, the incidence decreased in the ADM group to 3 cases (4.6%), and no cases (0%) were reported in the PU group (Table 2).

**Table 2.** Complications After Prepectoral Breast Reconstruction in the 2 Groups

Median (q1-q3)/*n* (%)	Overall (*n* = 135)	PU (*n* = 70)	ADM (*n* = 65)	*P*-value
Seroma (pre 1 month)	24 (17.8)	2 (2.9)	22 (33.8)	<.001
Hematoma (pre 1 month)	1 (0.7)	0 (0.0)	1 (1.5)	.482
Wound dehiscence (pre 1 month)	8 (5.9)	1 (1.4)	7 (10.8)	.029
Infection (pre 1 month)	5 (3.7)	1 (1.4)	4 (6.2)	.195
Implant extrusion	0 (0.0)	0 (0.0)	0 (0.0)	—
RBS	3 (2.2)	2 (2.9)	1 (1.5)	1.000
Severe redness	7 (5.2)	1 (1.4)	6 (9.2)	.055
Discomfort (post 1 month)	25 (18.5)	8 (11.4)	17 (26.2)	.045
Seroma (post 1 month)	3 (2.2)	0 (0.0)	3 (4.6)	.109
Hematoma (post 1 month)	0 (0.0)	0 (0.0)	0 (0.0)	—
Infection (post 1 month)	10 (7.4)	3 (4.3)	7 (10.8)	.195
BIA-ALCL	0 (0.0)	0 (0.0)	0 (0.0)	—
Discomfort (post 1 month)	24 (17.8)	10 (14.3)	14 (21.5)	.368
Capsular contracture 3 years				<.001
1a	33 (24.4)	27 (38.6)	6 (9.2)	
1b	44 (32.6)	26 (37.1)	18 (27.7)	
2	41 (30.4)	9 (12.9)	32 (49.2)	
3	15 (11.1)	8 (11.4)	7 (10.8)	
4	1 (0.7)	0 (0.0)	1 (1.5)	
Capsular contracture 5 years				<.001
1a	18 (13.3)	15 (21.4)	3 (4.6)	
1b	29 (21.5)	21 (30.0)	8 (12.3)	
2	39 (28.9)	17 (24.3)	22 (33.8)	
3	38 (28.1)	11 (15.7)	27 (41.5)	
4	10 (7.4)	6 (8.6)	4 (6.2)	
Worsening between 3 and 5 years	80 (59.3)	41 (58.6)	39 (60.0)	.780
Minus areas/adhesions	11 (8.1)	5 (7.1)	6 (9.2)	.658
Thinning/redness	12 (8.9)	5 (7.1)	7 (10.8)	.459
Step-off deformity	28 (20.7)	14 (20.0)	14 (21.5)	.826
Rippling grade				.830
1	65 (48.1)	32 (45.7)	33 (50.8)	
2	43 (31.9)	23 (32.9)	20 (30.8)	
3	22 (16.3)	12 (17.1)	10 (15.4)	
4	4 (3.0)	3 (4.3)	1 (1.5)	
Explantation	4 (3.0)	0 (0.0)	4 (6.2)	.051

ADM, acellular dermal matrix; BIA-ALCL, breast implant-associated anaplastic large cell lymphoma; PU, polyurethane; RBS, red breast syndrome.

#### Infection

The infection rate in the PU group was observed in 1 case (1.4%), compared with 4 cases (6.2%) in the ADM group. All infections were treated with antibiotics; however, 1 patient in the ADM group required implant removal due to an unmanageable infection. An increase in infection rates was observed 1 month after surgery in both groups, although it remained lower in the PU group (3 cases, 4.3%) compared with the ADM group (7 cases, 10.8%; Table 2).

#### Hematoma

No hematomas were recorded in the PU group. One hematoma was observed in the ADM group, managed conservatively without the need for implant removal. At medium-term follow-up, no hematomas were reported in either group (Table 2).

#### Red Breast Syndrome

Red breast syndrome (RBS) was observed in 1 case (1.5%) in the ADM group and 2 cases (2.9%) in the PU group. This phenomenon was temporary and resolved with conservative treatment.

No cases of implant extrusion were recorded in either group (Table 2).

### Long-Term Complications

#### Capsular Contracture

Capsular contracture of Grades 3 and 4 was evaluated at the 3-year follow-up. In the PU group, 8 cases (11.4%) were recorded, all classified as Grade 3. Similarly, in the ADM group, 8 cases (12.3%) were observed, including 1 case of Grade 4. At the 5-year follow-up, capsular contracture was observed in 17 cases (24.3%) in the PU group and 31 cases (47.7%) in the ADM group (Table 2). Most cases of capsular contracture in the ADM group occurred in the absence of PMRT. Specifically, in the PU group, there were 48 breasts not subjected to PMRT, of which only 2 cases (4.2%) developed Grade 3 capsular contracture after 5 years. Conversely, in the ADM group, there were 62 breasts not subjected to PMRT, of which 30 cases (47.4%) developed pathological capsular contracture (Table 3). As described in Table 3, reconstructions with ADM have an 89% higher risk (OR 1.893, 95% CI, 0.728-5.086), adjusting for confounding, of worsening capsular contracture, although this was not statistically significant (*P* = .20). Overall, even if not statistically significant (*P* = .75), undergoing PMRT increases the risk of developing pathological capsular contracture by 197% (OR 2.969, 95% CI, 0.954-10.825).

**Table 3.** Characteristics of Capsular Contracture in Breasts With and Without PMRT Between the 2 Groups

Capsular contracture	Overall	PU	ADM	*P*-value
PMRT	*n* = 25	*n* = 22	*n* = 3	
3 Years postop				.409
1a	3 (12.0)	2 (9.1)	1 (33.3)	
1b	6 (24.0)	5 (22.7)	1 (33.3)	
2	8 (32.0)	7 (31.8)	1 (33.3)	
3	8 (32.0)	8 (36.6)	0 (0.0)	
4	0 (0.0)	0 (0.0)	0 (0.0)	
5 Years postop				.236
1a	2 (8.0)	1 (4.5)	1 (33.3)	
1b	3 (12.0)	2 (9.1)	1 (33.3)	
2	4 (16.0)	4 (18.2)	0 (0.0)	
3	10 (40.0)	9 (40.9)	1 (33.3)	
4	6 (24.0)	6 (27.3)	0 (0.0)	
Worsening between 3 and 5 years	17 (68.0)	16 (72.3)	1 (33.3)	.231
No PMRT	*n* = 110	*n* = 48	*n* = 62	
3 Years postop				<.001
1a	30 (27.3)	25 (52.1)	5 (8.1)	
1b	38 (34.5)	21 (43.8)	17 (27.4)	
2	33 (30.0)	2 (4.2)	31 (50.0)	
3	7 (6.4)	0 (0.0)	7 (11.3)	
4	1 (0.9)	0 (0.0)	1 (1.6)	
5 Years postop				<.001
1a	16 (14.5)	14 (29.2)	2 (3.2)	
1b	26 (23.6)	19 (39.6)	7 (11.3)	
2	35 (31.8)	13 (27.1)	22 (35.5)	
3	28 (25.5)	2 (4.2)	26 (41.9)	
4	4 (3.6)	0 (0.0)	4 (6.5)	
Worsening between 3 and 5 years	63 (57.3)	25 (52.1)	38 (61.3)	.284

Multiple logistic model adjusted for age, BMI, mastectomy volume, implant volume, mastectomy, flap thickness, to assess the association between worsening capsular contracture (3-4 vs 1-2) and ADM, PMRT. ADM, acellular dermal matrix; PMRT, postmastectomy radiotherapy; PU, polyurethane.

In cases of bilateral breast reconstruction in no PMRT patients, the degree of capsular contracture differed between the 2 breasts in patients who experienced complications, such as seroma (0 of 3), infection (2 of 6), or dehiscence (1 of 5; Supplemental Table 1). Additionally, among bilateral reconstructions without complications, disparities in capsular contracture between the 2 breasts were observed in some cases: 4 out of 9 in the ADM group and 4 out of 12 in the PU group (Supplemental Table 1).

#### Rippling and Step-Off Deformity

Grades 3 and 4 of rippling occurred with an incidence of 15 cases (21.4%) in the PU group and 11 cases (16.9%) in the ADM group (Table 2). The degrees of rippling were not associated with the measurements of mastectomy flap thickness (Grades 1 and 2), BMI, age, and the volumes of the implants and mastectomy in either group (Supplemental Table 2).

Step-off deformity was observed in 14 cases (20%) in the PU group and 14 cases (21.5%) in the ADM group. Mastectomy flap thickness was evaluated, revealing a median thickness of 1.0 mm (IQR, 0.9-1.2 mm) overall, with a significantly higher median thickness in the PU group (1.10 mm; IQR, 0.9-1.2 mm) compared with the ADM group (1.0 mm; IQR, 0.8-1.0 mm; *P* < .001). Additionally, flap thickness ≥1 mm was more frequent in the PU group (74.3%) compared with the ADM group (64.6%; Table 1). To date, no cases of BIA-ALCL have been observed in either group (Table 2).

### Aesthetic Outcomes

The evaluation of aesthetic outcomes demonstrated good aesthetic results for PU, with significantly different performance compared with ADM in terms of breast symmetry/position (*P* = .03) and overall aesthetic result (*P* = .02). However, minor surgical procedures are necessary to improve aesthetics and symmetry in 41 cases overall (42.3%; Table 4). A significant association was observed between the type of reconstruction group and the overall aesthetic outcome, with a 63% reduction in the risk of being in a better aesthetic category in ADM compared with PU, after adjusting for confounding factors (OR 0.372, 95% CI, 0.158-0.856; *P* = .023).

**Table 4.** Description of Aesthetic Outcomes in the 2 Groups and Their Association With ADM, Through Ordinal Logistic Models Adjusted for Age, BMI, and Intervention in Both Breasts

*n* (%)	Overall (*n* = 97)	PU (*n* = 54)	ADM (*n* = 43)	OR (95% CI)	*P*-value
Volume				0.597 (0.258-1.363)	.222
Marked discrepancy	9 (9.3)	5 (9.3)	4 (9.3)		
Moderate discrepancy	44 (45.4)	21 (38.9)	23 (53.5)		
Symmetric	43 (44.3)	28 (51.9)	15 (34.9)		
Contour				0.581 (0.252-1.323)	.197
Marked deformity	8 (8.2)	4 (7.4)	4 (9.3)		
Moderate deformity	40 (41.2)	19 (35.2)	21 (48.8)		
Little/no deformity	48 (49.5)	31 (57.4)	17 (39.5)		
Inframammary crease				0.885 (0.333-2.381)	.806
Poorly defined	2 (2.1)	1 (1.9)	1 (2.3)		
Demarcated, asymmetric	23 (23.7)	12 (22.2)	11 (25.6)		
Well defined, symmetric	71 (73.2)	41 (75.9)	30 (69.8)		
Breast symmetry/breast position				0.676 (0.290-1.571)	.343
Marked discrepancy	6 (6.2)	5 (9.3)	1 (2.3)		
Mild discrepancy	37 (38.1)	15 (27.8)	22 (51.2)		
Little/no discrepancy	53 (54.6)	34 (63.0)	19 (44.2)		
Breast symmetry/breast projection				0.844 (0.359-1.989)	.686
Marked discrepancy	4 (4.1)	3 (5.6)	1 (2.3)		
Mild discrepancy	37 (38.1)	18 (33.3)	19 (44.2)		
Little/no discrepancy	55 (56.7)	33 (61.1)	22 (51.2)		
Breast symmetry/breast Shape				0.422 (0.181-0.963)	.042
Marked discrepancy	9 (9.3)	5 (9.3)	4 (9.3)		
Mild discrepancy	40 (41.2)	17 (31.5)	23 (53.5)		
Little/no discrepancy	47 (48.5)	32 (59.3)	15 (34.9)		
Overall result				0.372 (0.158-0.856)	.021
Poor/needing further operations,	8 (8.2)	4 (7.4)	4 (9.3)		
Fair/needing second minor surgery	41 (42.3)	17 (31.5)	24 (55.8)		
Good/definitive results	47 (48.5)	33 (61.1)	14 (32.6)		

ADM, acellular dermal matrix; OR, odds ratio; PU, polyurethane.

## DISCUSSION

The authors of this study compare the outcomes of 2 widely used approaches in prepectoral breast reconstruction: PU-coated implants and implants with total ADM cover. The results provide valuable insights into the respective advantages and disadvantages of each technique, contributing to the ongoing debate on the best approach for prepectoral breast reconstruction.

### Complication Profile

**Figure 1.**
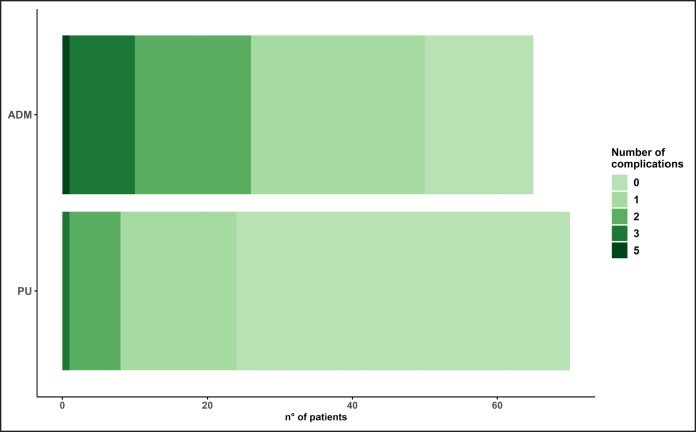
Graph showing the number of complications in the 2 groups (polyurethane-coated implants vs acellular dermal matrix–covered implants).

The findings demonstrate that PU-coated implants are associated with lower rates of early complications, including seroma and infection (Table 5, Figure 1).

**Table 5.** Number of Complications in Breasts Across the 2 Groups

Median (q1-q3)/*n* (%)	Overall (*n* = 135)	PU (*n* = 70)	ADM (*n* = 65)	*P*-value
Number of complication (<1 month + >1 month)				<.001
0	76 (56.3)	52 (74.3)	24 (36.9)	
1	26 (19.3)	10 (14.3)	16 (24.6)	
2	22 (16.3)	8 (11.4)	14 (21.5)	
3	9 (6.7)	0 (0.0)	9 (13.8)	
4	2 (1.5)	0 (0.0)	2 (3.1)	

ADM, acellular dermal matrix; PU, polyurethane.

The adhesive properties of the PU foam likely play a crucial role in reducing fluid accumulation, as the foam adheres firmly to surrounding tissues, limiting dead spaces where fluid could collect. The “Velcro” effect of the PU also helps to stabilize the implant, reducing the risk of displacement and rotation, and potentially explaining the lower rate of complications such as seroma and infection in Group A.

#### Seroma

The incidence of seroma represents one of the main differences between the 2 groups. In patients with PU implants, seroma cases stand at 2.9% in the early postoperative period and drop to 0% in the mid-term. In contrast, in the ADM group, the incidence is significantly higher: 33.8% in the early period and 4.6% in the mid-term.

This discrepancy suggests that, on one hand, the PU coating provides stronger adhesion to the tissues, reducing the spaces where fluid can accumulate. On the other hand, the ADM may encourage the formation of a more “humid” and inflammatory environment, likely due to increased local fluid exudation and the need for greater vascularization and tissue integration of the matrix itself. Moreover, seroma not only increases the risk of secondary infection but can also compromise implant integration, prolong recovery times, and sometimes necessitate additional procedures (such as ultrasound-guided aspirations or, in more severe cases, surgical reintervention).

#### Infection and Red Breast Syndrome

Despite ADM fostering anatomical integration that more closely resembles natural tissues, the ADM group showed an overall higher infection rate compared with the PU group. It is plausible that the bacterial colonization process, combined with a larger biologically active surface area, increases the risk of inflammation. In some cases, this presents as RBS, characterized by transient redness and edema which, although generally self-limiting, can sometimes require a more aggressive pharmacological approach.

It is important to note that PU-coated implants retain the antibiotic solution within the sponge-like structure of the coating, whereas in textured implants, the antibiotic often tends to run off. This mechanism may partly explain the lower risk of infection observed in the PU group.

#### Capsular Contracture

**Figure 2.**
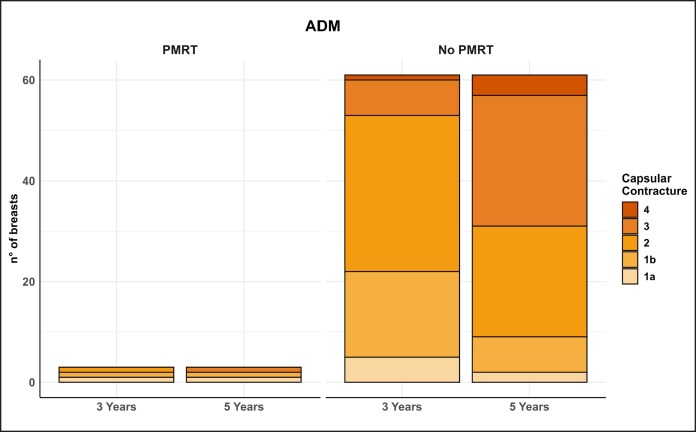
Graph illustrating the progression of capsular contracture at 3 and 5 years in the acellular dermal matrix group, with and without postmastectomy radiotherapy.

**Figure 3.**
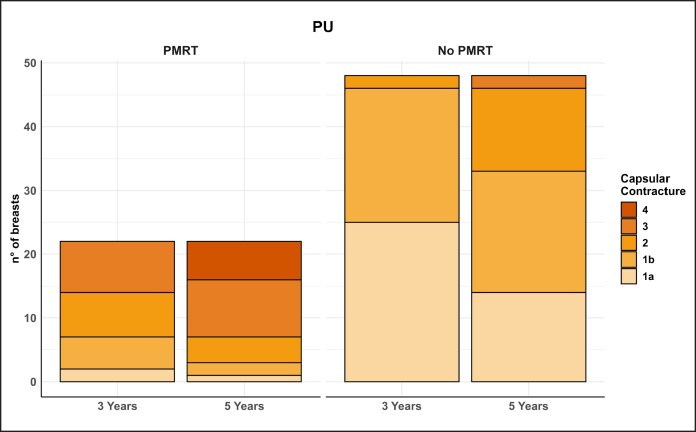
Graph illustrating the progression of capsular contracture at 3 and 5 years in the polyurethane group, with and without postmastectomy radiotherapy.

**Figure 4.**
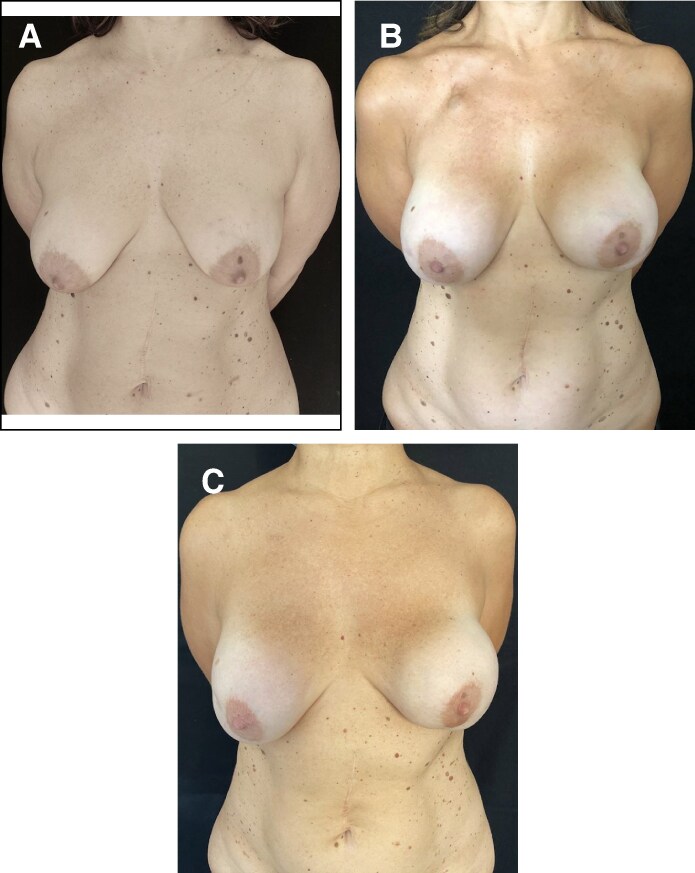
Photographs depicting a 45-year-old female patient who underwent bilateral breast reconstruction with acellular dermal matrix and textured implants. The patient did not undergo postmastectomy radiotherapy, and no complications were observed in the short-term postoperative period. (A) Preoperative view. (B) Postoperative view at 3 years; right breast capsular contracture Grade 1a, left breast capsular contracture Grade 3. (C) Postoperative view at 5 years shows progression of the capsular contracture on the left breast (right breast capsular contracture Grade 1b, left breast capsular contracture Grade 4).

One of the most significant aspects emerging from this study is the lower incidence of capsular contracture associated with PU-coated implants. This finding confirms evidence from the literature suggesting that PU-coated implants may offer intrinsic protection against the development of capsular contracture.^9^ The data further suggest that ADM reconstructions develop more severe contracture in the absence of PMRT, indicating that ADM may elicit a more intense and prolonged inflammatory reaction compared with PU-coated implants in the long term (Table 3, Figures 2-4).

The PU foam coating appears to reduce implant migration into the pocket due to its ability to adhere tightly to surrounding tissues. This stability minimizes irritation and micro-movements that could trigger a chronic inflammatory response and, consequently, excessive fibrosis. Additionally, the PU foam seems to influence the formation of the periprosthetic capsule, making it less dense and less prone to contracture.

The data regarding capsular contracture are inconsistent with the current literature, and this may be due to the extreme subjectivity of the clinical examination used for the Baker–Spear classification, despite our efforts to apply it with the utmost precision. To effectively compare the formation, quality, and contracture of the periprosthetic capsule, we should probably rely on imaging studies.^25^ However, although the data are not in line with current literature, the authors of this study highlighted a higher degree of capsular contracture at 5 years in the group undergoing reconstruction with ADM, even in the absence of PMRT.

### Aesthetic Outcomes

**Figure 5.**
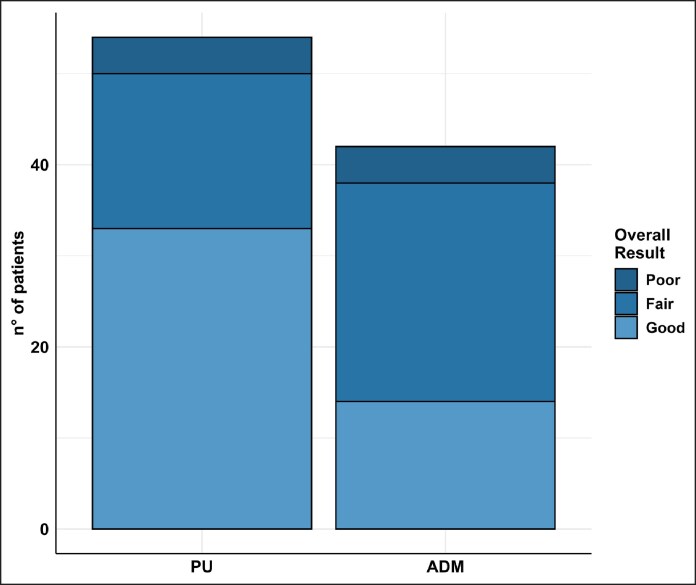
Graph illustrating the overall aesthetic outcomes in both groups.

**Figure 6.**
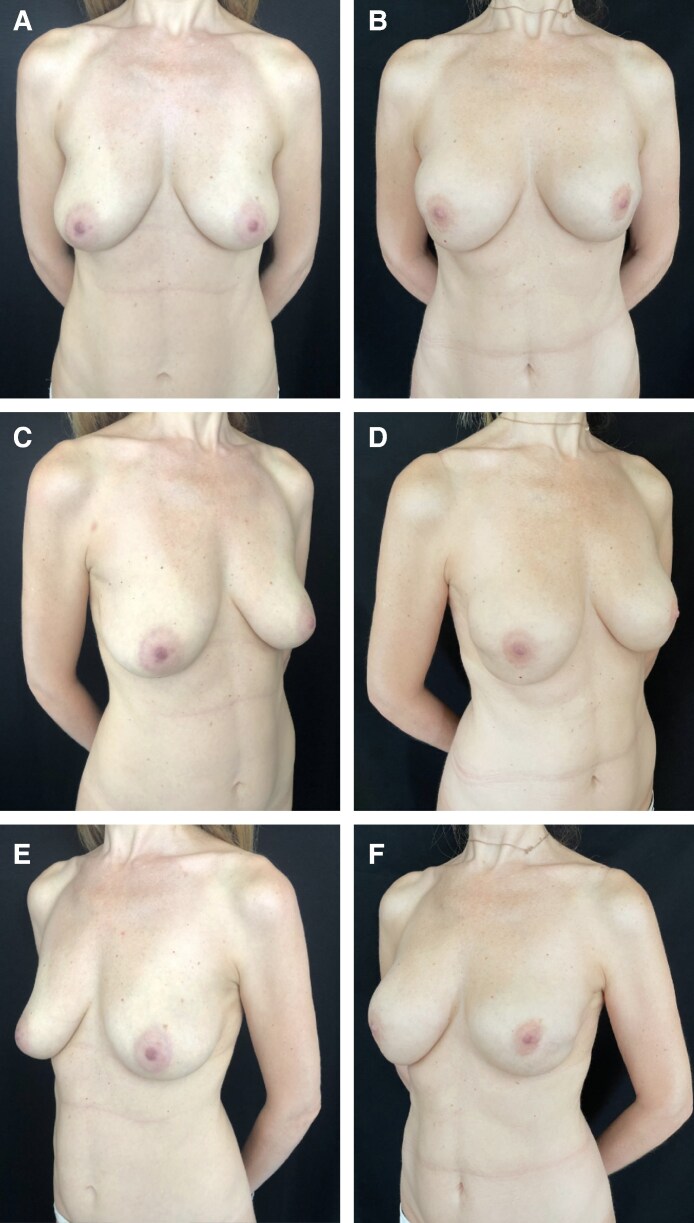
Photographs depicting a 47-year-old female patient who underwent bilateral breast reconstruction with polyurethane-coated implants. The patient did not undergo postmastectomy radiotherapy and no complications were observed in the short-term postoperative period. Preoperative (A) frontal view and (C, E) 3/4 views. Postoperative (B) frontal view and (D, F) 3/4 views 5 years postoperatively. The right breast exhibits capsular contracture Grade 1b, and the left breast exhibits capsular contracture Grade 1b.

**Figure 7.**
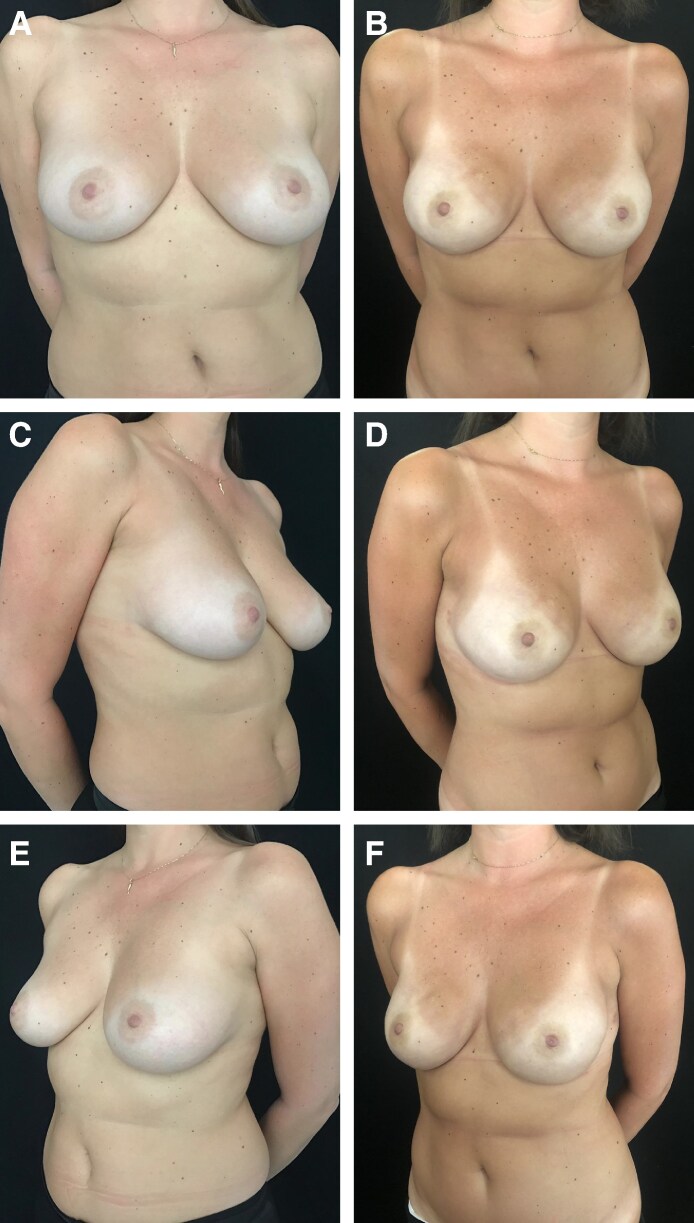
Photographs of a 38-year-old female patient who underwent bilateral breast reconstruction with polyurethane-coated implants. The patient received postmastectomy radiotherapy in the right breast, and no complications were observed in the short-term postoperative period. Preoperative (A) frontal view and (C, E) 3/4 views. Postoperative (B) frontal view and (D, F) 3/4 views 4 years postoperatively. The right breast exhibits capsular contracture Grade 1b and left breast capsular exhibits contracture Grade 1a.

**Figure 8.**
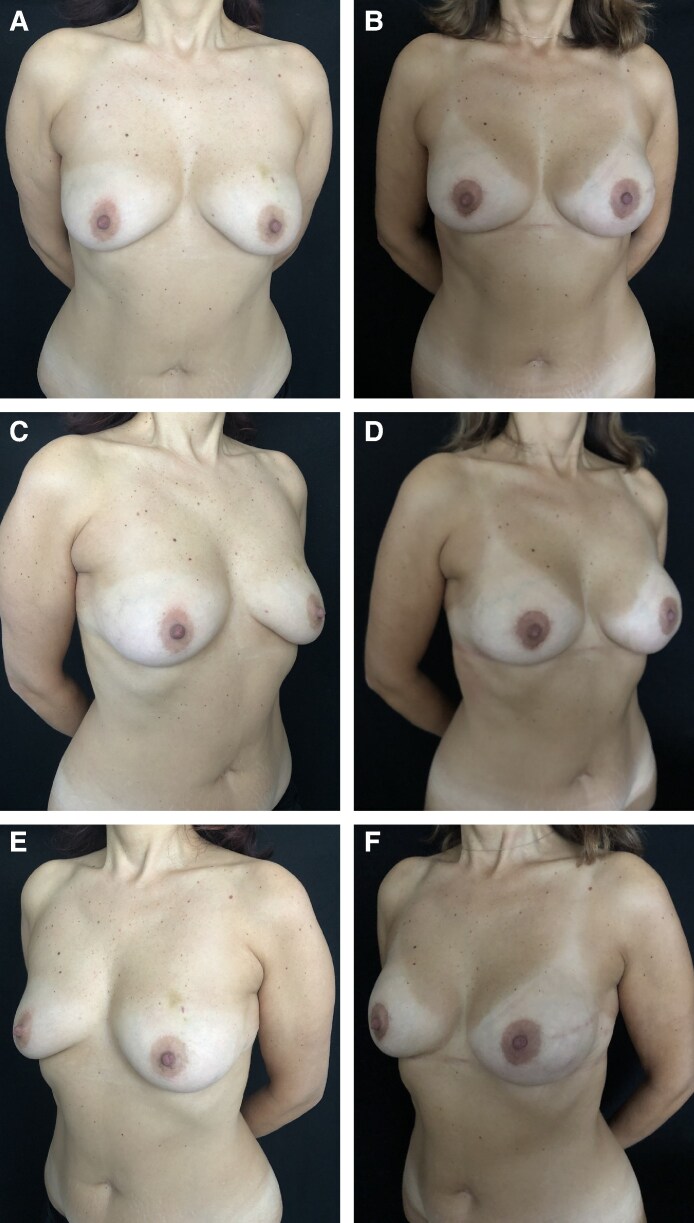
Photographs of a 50-year-old female patient who underwent reconstruction of the left breast with polyurethane-coated implants and right breast symmetrization mastopexy. The patient did not receive postmastectomy radiotherapy, and no complications were observed in the short-term postoperative period. Preoperative (A) frontal view and (C, E) 3/4 views. Postoperative (B) frontal view and (D, F) 3/4 views 5 years postoperatively. The left breast exhibits capsular contracture Grade 1a.

The aesthetic outcomes, particularly regarding “rippling” and implant visibility, were slightly better in the ADM group, albeit without statistical significance. This is consistent with the described benefits of ADM in providing a more natural contour to the reconstructed breast, especially in patients with thin skin flaps. ADM should effectively mask the edges of the implant, reducing the risk of visible rippling, a known concern with PU-coated implants. Nevertheless, we observed that the overall result and breast shape were superior in the PU group reconstructions over time, likely due to the higher incidence of capsular contracture in the ADM group (Figure 5). However, the trade-off between better aesthetic results and higher complication rates must be carefully considered when selecting the most appropriate reconstructive technique for each patient (Figures 6, 7, 8). With regard to the type of procedure performed (NSM, SSM, and SRM), it likely influenced the aesthetic assessments, particularly in terms of symmetry and volume. Nevertheless, the sample of patients who underwent SSM and SRM is very small, especially when stratified by implant type; therefore, any comparative evaluation would not be statistically significant.

This study has several limitations that must be considered when interpreting the results. First, the retrospective and observational design introduces the potential for selection bias, as evidenced by the unequal distribution of risk factors between the groups. Additionally, this design limits the ability to establish causal relationship. Second, although statistical analyses were adjusted for potential confounding variables, such as age, BMI, mastectomy volume, implant volume, and flap thickness, unmeasured confounders may still influence the observed outcomes. Third, the relatively small sample size, particularly for certain subgroups (eg, patients undergoing PMRT), may reduce the power to detect significant associations. Fourth, the data were collected from a single institution, which may limit the generalizability of the findings to other settings or patient populations. Finally, the potential correlation between the 2 breasts of the same individual was not accounted for in the analysis due to the small sample size and the assumption of independence in the performance of each breast within the same person. This simplification may have introduced bias, as outcomes from the same individual are likely interdependent.

## CONCLUSIONS

The findings of this study suggest that PU-coated implants provide a more favorable safety profile, characterized by a lower incidence of seromas and infections, a reduced long-term frequency of capsular contracture, and a more aesthetically pleasing outcome. Conversely, complete coverage with ADM—while demonstrating an improvement in breast contour definition, particularly in cases of rippling and step-off deformity—is associated with a higher risk of fluid collections (seromas) and infections, as well as increased severity of capsular contracture in most cases.

The choice of the optimal prepectoral reconstructive technique should therefore be personalized, considering the patient's anatomical characteristics (such as flap thickness, implant volume, and any risk factors) and the desired aesthetic and functional outcomes. Larger prospective multicenter studies with extended follow-up periods will offer more detailed insights, aiding surgeons in developing increasingly targeted treatment algorithms for the growing population of women who choose immediate implant-based reconstruction. Certainly, the prevention and management of seromas remain among the most crucial factors in achieving long-lasting, complication-free results.

## Supplemental Material

This article contains supplemental material located online at https://doi.org/10.1093/asj/sjaf158.

## Supplementary Material

sjaf158_Supplementary_Data
