# Supplementary material for: Prepectoral Breast Reconstruction: Early and Long-Term Complications and Outcomes of Total Coverage Acellular Dermal Matrix and Implants Vs Polyurethane-Coated Implants Without Use of Acellular Dermal Matrix
Source: Aesthet Surg J. 2025 Aug 8;46(1):38–48. doi: 10.1093/asj/sjaf158 (PMC12706863; doi:10.1093/asj/sjaf158)
Supplement: sjaf158_Supplementary_Data [file sjaf158_supplementary_data.zip › Supplemental_Table_1.docx]

**Supplemental Table 1. Differences in capsular contracture between breasts in bilateral reconstruction without PMRT.**

**Number of patients with discordant capsular contracture, where one breast exhibits specific complications and the other does not**

|  | **Seroma** | **Dehiscence** | **Infection** |
| --- | --- | --- | --- |
| **ADM** | 0/3 | 1/4 | 2/4 |
| **PU** | 0 | 0/1 | 0/2 |

*Numbers expressed as: n° of patients with discordant capsular contractures/n° of patients per group

**Number of patients with discordant capsular contracture, where one breast exhibits any complications and the other does not**

|  | **No complications** | **Complications** |
| --- | --- | --- |
| **ADM** | 4/9 | 5/12 |
| **PU** | 3/9 | 1/3 |

*Numbers expressed as: n° of patients with discordant capsular contractures/n° of patients per group
