# Supplementary material for: Prepectoral Breast Reconstruction: Early and Long-Term Complications and Outcomes of Total Coverage Acellular Dermal Matrix and Implants Vs Polyurethane-Coated Implants Without Use of Acellular Dermal Matrix
Source: Aesthet Surg J. 2025 Aug 8;46(1):38–48. doi: 10.1093/asj/sjaf158 (PMC12706863; doi:10.1093/asj/sjaf158)
Supplement: sjaf158_Supplementary_Data [file sjaf158_supplementary_data.zip › Supplemental_Table_2.docx]

**Supplemental Table 2. Multiple logistic model* to assess the association between high rippling degree (3-4 vs 1-2) and ADM, thickness, age, BMI, mastectomy volume and implant volume.**

|  | **OR** | **IC 95%** | **p-value** |
| --- | --- | --- | --- |
| ADM | 1.256 | 0.413; 3.773 | 0.684 |
| Thickness: |  |  |  |
| <1 | Ref | Ref | Ref |
| ≥1 | 1.149 | 0.395; 3.614 | 0.804 |
| Age | 0.997 | 0.938; 1.057 | 0.925 |
| BMI | 0.874 | 0.712; 1.042 | 0.164 |
| Mastectomy volume | 1.000 | 0.995; 1.004 | 0.883 |
| Implant volume | 0.997 | 0.989; 1.005 | 0.502 |
